# Effectiveness of some plant extracts in biocontrol of induced onion basal rot disease in greenhouse conditions

**DOI:** 10.1186/s13568-024-01721-4

**Published:** 2024-06-14

**Authors:** Mohamed G. A. Hegazy, Abdel-Raddy M. Ahmed, Ahmed Fathy Yousef, Waleed M. Ali, Alyaa Nasr, Ezzat H. Elshazly, Mohamed E. Shalaby, Islam I. Teiba, Osama A. M. Al-Bedak

**Affiliations:** 1 Department of Agricultural Botany, Faculty of Agriculture, Al-Azhar University (Assiut Branch), Assiut, 71524 Egypt; 2 Department of Agronomy (Biochemistry), Faculty of Agriculture, Al-Azar University (Assiut Branch), Assiut, 71524 Egypt; 3 Department of Horticulture, Faculty of Agriculture, University of Al-Azhar (Assiut Branch), Assiut, 71524 Egypt; 4 Botany and Microbiology Department, Faculty of Science, Menoufia University, Shebin Elkom, 32511 Egypt; 5 Botany and Microbiology Department, Faculty of Science, Al-Azhar University (Assiut Branch), Assiut, 71524 Egypt; 6 Department of Plant Production, Faculty of Agriculture (Saba Basha), Alexandria University, Alexandria, 21531 Egypt; 7 Microbiology, Botany Department, Faculty of Agriculture, Tanta University, Tanta, 31527 Egypt; 8 Assiut University Mycological Centre, Assiut, 71511 Egypt; 9 ERU Science& Innovation Center of Excellence, Egyptian Russian University, Badr city, 11829 Egypt

**Keywords:** *Artemisia absinthium*, *Calotropis procera*, GC–MS analysis, *Moringa oleifera*, Secondary metabolites, *Syzygium aromaticum*

## Abstract

One of Egypt’s most notable and historically significant vegetable crops is the Liliaceae plant, *Allium cepa* L. In this study, the effectiveness of methanolic extracts of *Artemisia absinthium* leaves, *Calotropis procera* latex, *Moringa oleifera* seeds, and *Syzygium aromaticum* clove was investigated in vitro and, in a greenhouse, setting against *Fusarium oxysporum*, the pathogen that causes onion basal rot in Assiut Governorate, Egypt. The *S. aromaticum* extract exhibited the inhibition peak (63.3%), whereas the *A. absinthium* extract had the lowest inhibition impact against *F. oxysporum* growth (41.1%). The gas chromatography-mass spectroscopy (GC–MS) analysis revealed that 82 important compounds, with abundances ranging from low to high, were present in the tested *S. aromaticum*’s methanolic extract. The primary components were acetaldehyde, hydroxy- and 2-propanone, 1,1,3,3-tetrachloro-(42.71%), 1,2-ethanediol, and methyl alcohol (34.01%). In comparison to the infected control, the disease severity was significantly reduced by 20% with the use of a plant extracts mixture and Dovex 50% and increased by 62.22% with the use of an extract from *A. absinthium*. When compared to the infected control, onion plant fresh weight and dry weight were considerably higher under the clove extract therapy. The plant extracts used in this study’s testing contain a number of active ingredients, including amino acids, vitamins, minerals, antioxidants, and enzymes, which is probably why they have such positive impacts. The application of a combination of plant extracts was suggested as a feasible strategy for improving the growth and productivity of onion plants by the study’s findings. More research is needed to comprehend the mechanisms by which plant extracts promote plant development and to optimize the concentration and timing of administration.

**Supplementary Information:**

The online version contains supplementary material available at 10.1186/s13568-024-01721-4.

## Introduction

One of the world’s most important bulb crops, the onion (*Allium cepa*), is regarded as one of the most important commercial vegetable crops in the world including Egypt (Hussein et al. 2014). It is one of the oldest vegetables that has been used as spice and in medicine for thousands of years (Keusgen 2002). It is a rich source of carbohydrates and minerals including protein, vitamin C, phosphorus and calcium (Chakraborty et al. 2022), as well as has several medicinal uses (Gupta et al. 2015). In the field and in storage, several pathogens attack onions, lowering their quality and yield (Khalifa et al. 2016). The basal rot of onions caused by *Fusarium oxysporum* holds economic significance due to its impact, resulting in the decay of the basal plate of the bulb, further infection of the bulb scales, and causing the most significant losses during storage. Several *Allium* species, including chive, garlic, and shallot, as well as onion, are affected by *F. oxysporum*, which causes *Fusarium* basal rot.

Onion bulbs and shallots are susceptible to *Fusarium* basal rot of onion at all stages of growth (Cramer 2000), which is a disease with significant economic impact. According to Bayraktar and Dolar (2011), the fungi *F. oxysporum*, *F. solani*, *F. acuminatum*, *F. culmorum*, *F. equiseti*, *F. proliferatum*, *F. subglutinans*, *F. redolens*, and *F. tricinctum* can all cause basal rot disease of onions. For managing fungal diseases in crops, fungicides, extended rotations, and resistant cultivars have been used. Because several of them have been associated with increased rates of a number of cancers and are regarded to pose significant health hazards, using synthetic fungicides is not environmentally friendly (Beckerman et al. 2023; Rongai et al. 2015). Antimicrobial effects of medicinal plants have long been established. Plant extracts may be the ideal treatment because they are straightforward to test in vitro (Dissanayake 2014). Alkaloids, flavonoids, phenolic compounds, and tannins are a few of the chemical substances that can be found in medicinal plants. Alkaloids, a class of organic compounds containing nitrogen, are recognized in phytochemical research for their potential antibacterial properties, distinct from phenolic compounds (Dubale et al. 2023).

*Syzygium aromaticum*, or cloves (Family: *Myrtaceae*) are unopened, fragrant, dried flower buds that are native to India, Indonesia, Zanzibar, Mauritius, and Ceylon. It has been documented that the herbal plants demonstrated antifungal and anti-mycotoxigenic actions in addition to having potential antioxidant properties (Dikhoba et al. 2019). Additionally, *S. aromaticum* methanol extract displayed remarkable antibacterial effectiveness against *Bacillus subtilis*, *Pseudomonas aeruginosa*, and *Staphylococcus aureus* (Vizhi et al. 2016). In tropical areas of Asia, Africa, and South America, the Moringa plant (*Moringa oleifera*) is frequently farmed (Sato et al. 2013). The leaves, roots, and bark of the Moringa tree have been demonstrated coagulant and antibacterial properties (Taiwo et al. 2020), as well as being useful for treating water (Sato et al. 2013). *Staphylococcus aureus*, *Staphylococcus epidermidis*, *Streptococcus mitis*, *Streptococcus pneumoniae*, *Enterococcus faecalis*, *Escherichia coli*, and *Legionella pneumophila* can all be successfully treated with *M. oleifera* seed extract and recombinant protein.

*Calotropis procera* (Family *Asclepiadaceae*) is also known as milk weeds due to the latex it produces, and it is distributed in several regions of the world (Kumar 2015). With the help of the powerful medication *C. procera*, leucoderma, leprosy, ulcers, tumors, piles, and disorders of the spleen, liver, and abdomen can all be effectively treated. The plant has undergone phytochemical analysis for cardenolides, anthocyanins, hydrocarbons, and triterpenoids, and it has been shown to exhibit a number of pharmacological properties including cardiac tonic, hepatoprotective, antibacterial, as well as anticancer (Ahmed et al. 2004; Karela 2023). According to reports, the plant’s latex has purgative effects (Karela 2023; Rajesh et al. 2005), procoagulant activity and wound healing activity (Nalwaya et al. 2009; Obagu and Ajiboso 2023), antibacterial activity (Ibrahim et al. 2023; Kumar et al. 2010), and anti-inflammatory and anti-oxidant activity.

*Artemisia absinthium* L., (wormwood), is a significant perennial shrubby medicinal plant that is indigenous to Asia, the Middle East, Europe, and North Africa, and belongs to the family *Asteraceae* (Beigh and Ganai 2017; Sharopov et al. 2012). It was a common Greek social prescription throughout the ancient era and served as an antiseptic, antimalarial, anthelmintic, antioxidant, hepatoprotective, antipyretic, anti-breast cancer, and neuroprotective. Research has shown that several *Artemisia* species, including *A. kulbadica*, *A. sieberi*, *A. turanica*, *A. santolina*, and *A. diffusa*, have deadly effects on human Caucasian hepatocyte carcinoma (HepG-2) and human Caucasian larynx cancer (Hep-2) cell lines (Emami et al. 2009). Consequently, for determination of the antifungal activity of *Syzygium aromaticum*, *Artemisia absinthium*, *Moringa oleifera*, and *Calotropis procera* methanol extracts against the pathogenic *F. oxysporum*, the responsible pathogen for onion basal rot, this study was designed.

## Materials and methods

### Isolation and identification of the pathogen

Using the direct plating method, the pathogenic fungus that causes the disease was isolated on potato dextrose agar (PDA) (Smith and Onions 1994). The diseased onion roots were placed on PDA-containing Petri plates, and the plates were then incubated at 25 °C until fungal growth was observed. The single spore isolation technique was used to create pure cultures from the generated colonies of the pathogenic fungal isolates. The pure culture of the pathogen was preserved as frozen mycelia stored at − 86 °C and lyophilized cultures, and it is available in the culture collection of the Assiut University Mycological Centre as AUMC 15798, as well as on cotton balls (Al-Bedak et al. 2019).

The process for molecular identification of the pathogen involved extracting fungal DNA using the procedures outlined by Moubasher et al. (2019), followed by PCR experiments conducted with SolGent EF-Taq at SolGent Company (SolGent Co., Ltd.; Daejeon, South Korea). The ITS region was amplified using the universal primers ITS1 and ITS4 (White et al. 1990). DNASTAR (version 5.05) was used to generate contiguous sequences of the Fusarium isolate from this study. The most similar sequences in GenBank database in addition to the Fusarium sequence in this study and the sequence of *Nectria dematiosa* CBS 126570 (as outgroup) were all aligned by Katoh and Standley (2013) applying the default option, and optimized by Criscuolo and Gribaldo (2010). MEGA X (version 10.2.6) was used to conduct maximum-likelihood (ML) and maximum-parsimony (MP) phylogenetic analyses (Kumar et al. 2018), and the robustness of the most parsimonious trees was evaluated by 1000 replications (Felsenstein 1985). Utilizing Modeltest 3.7’s Akaike Information Criterion (AIC), the optimum nucleotide substitution model for ML analysis was identified (Posada and Crandall 1998).

### Pathogenicity test

In the greenhouse at the Department of Plant Pathology, Faculty of Agriculture, Assiut University, Egypt, during the growing season 2022–2023, the pathogenicity test was conducted on 60-day-old onion seedlings (Giza Sabeeni cultivar). Four sets of plastic pots (each measuring 25 cm in diameter) were employed, one set serving as a negative control. Each container was filled with 3 kg of pre-sterilized sand: clay soil (1:2) before the five onion seedlings were planted. *Fusarium oxysporum* AUMC 15798 was cultured to create the inoculum, which was subsequently incubated for 21 days at 25 °C in Erlenmeyer conical flasks containing a 1:1 mixture of barley and sand. Seven days before planting the seedlings,, 3% (w/w) of the pathogen’s inoculum was mixed with the soil. After 120 days of the trial, when the percentage of disease severity in each treatment was noted, the basal rot disease grading scale was as follows:

1. Symptoms-free.
2. Up to 10% rotted roots.
3. 10–30% rotted roots with up to 10% rotted basal plates.
4. Completely rotted roots and 10–30% rotted basal plates.
5. Completely rotted roots and more than 30% rotted basal plate.

### Preparation of methanolic extracts

All plant components were cleaned and dried at 37 °C before being extracted with methanol. These dried plant components were then ground into a fine powder. Over 100 g of air-dried powder was used in each batch, which was macerated in 1000 mL of methanol following the method outlined by Handa (2008). The batches were left overnight at 37 °C in a shaking incubator. The methanol was evaporated at reduced pressure to collect the residuals for use in chemical analysis.

### Detection of different secondary metabolites in the plant extracts

A fraction of the aqueous filtrate from each plant extract was mixed with 5 mL of diluted ammonia solution, and then concentrated H_2_SO_4_ were added. This procedure was used to identify flavonoids. Each extract had a yellow color that showed flavonoids were present. For detection of tannins, 5 mL of distilled water, 2.5 g of the plant extract were dissolved, filtered, and the filtrate was then mixed with ferric chloride reagent, tannins were assumed to be present if a blue–black, green, or blue-green precipitate formed. Saponins were detected by boiling 2 g of the powdered sample in 20 mL of distilled water and then filtered. 10 mL of the filtrate was mixed with 5 mL of distilled water and shaken vigorously. The frothing was mixed with 3 drops of olive oil and shaken vigorously, then observed for the formation of emulsion (Mathew et al. 2012; Osman et al. 2022).

### Determination of total phenolic compounds (TPCs)

After refluxing with 30 mL of methanol containing 1.0% HCl for 10 min and centrifugation at 5000 rpm for 10 min, the total phenolic compounds were separately extracted from the plant extracts (0.5 g). According to a conventional procedure, the amount of total phenolic compounds present in the methanolic extracts is quantified as gallic acid equivalents (Maurya and Singh 2010).

### In vitro effect of methanolic extracts on the pathogen’s radial growth and determination of the minimal inhibitory concentration (MIC)

The residues from the four studied plants’ methanolic extracts were separately dissolved in DMSO at 100, 150, and 200 ppm. 1.0 mL of each concentration was separately mixed with 20 mL PDA in sterilized Petri dish to obtain 5, 7.5, and 10 ppm/plate. The plates were inoculated with a 6 mm diameter discs obtained from 4-day-old culture of *F. oxysporum*. The plates were subsequently incubated at 25 °C for 96 h. The percentage of radial colony growth suppression against control was then calculated according the following Equation, and the MIC was determined (Shivapratap et al. 1996).

$${\text{Radial}}\;{\text{growth}}\;{\text{inhibition}}\left( \% \right) = (D_{1} - D_{2} )/D_{1} \times 100$$

where D_1_, Colony diameter in control plate and D_2_, Colony diameter in treated plate.

### Gas chromatography–mass spectrometry (GC–MS) analysis

The methanolic extract of clove (whole plant) was prepared according to Wilson et al. (1995). In order to identify the active ingredients, the most potent methanol extract of *S. aromaticum* was analyzed using GC–MS. The analysis was conducted at the Chemistry Department, Faculty of Science, Assiut University. Using a fused-silica capillary column, the previously prepared supernatant was separated (DB-5MS: 30 mm 0.25 mm 0.25 m, Agilent J & W Scientific, Folsom, CA, USA). A steady flow rate of 1 mL per minute was used for high-purity (> 99.999%) helium as a carrier gas. 1 µL of the sample was injected at a final temperature of 300 °C after the oven’s initial temperature of 50 °C for 4 min. A temperature of 250 °C was chosen for the injection. For a total runtime of 75 min, EI-ionization was set at 70 eV. By comparing mass spectra of chemical compounds to the MAINLIB and replib databases, chemical compositions were deduced. The mass spectrum of unknown components was compared to that of recognized components based on name, structure, molecular weight, intensities, and fragmented ions. All internal standards and any pseudo-positive peaks were eliminated from the ‘data array’. The total peak area of each sample was used to calibrate the normalization process.

### Greenhouse experiment

T1 = methanolic extract of *C. procera* latex; T2 = methanolic extract of *A. absinthium* leaves; T3 = methanolic extract of *M. oleifera* seeds; T4 = methanolic extract of *S. aromaticum* clove; T5 = T_1_ + T_2_ + T_3_ + T_4_; T6 = methanolic extract of Dovex 50%; T7 = methanolic extracts of Dovex 50% + T_5_; T8 = negative control; T9 = positive control. In addition, Dovex 50% (Azoxystrobin 20% + Tebuconazole 30%) was used at the rate of 25 cm^3^ 100 L^−1^) as a reference for comparison.

### Determination of onion morphological parameters

120 days after planting the seedlings, the growth parameters were evaluated. The length of the shoots and roots was measured from the base of the bulb to the top of the plant using a centimeter ruler. The diameter of the bulb and neck were measured with digital calipers in millimeters. The fresh and dry mass was determined by weighing using a precise electronic balance with a sensitivity of 0.001 g. The freshly harvested shoots and roots were placed in paper bags and subjected to a drying process in an oven set at 75 ℃ for a minimum of 48 h to obtain the dry weight.

### Statistical analysis

One-way analysis of variance (ANOVA), was performed using the software (Statistix 8.1) for statistical analysis and assess for significance (Kim 2014). To look at the variations between the means that were statistically significant, Duncan’s multiple range tests with 95% confidence were performed.

## Results

### Isolation and identification of the pathogen

**Fig. 1:**
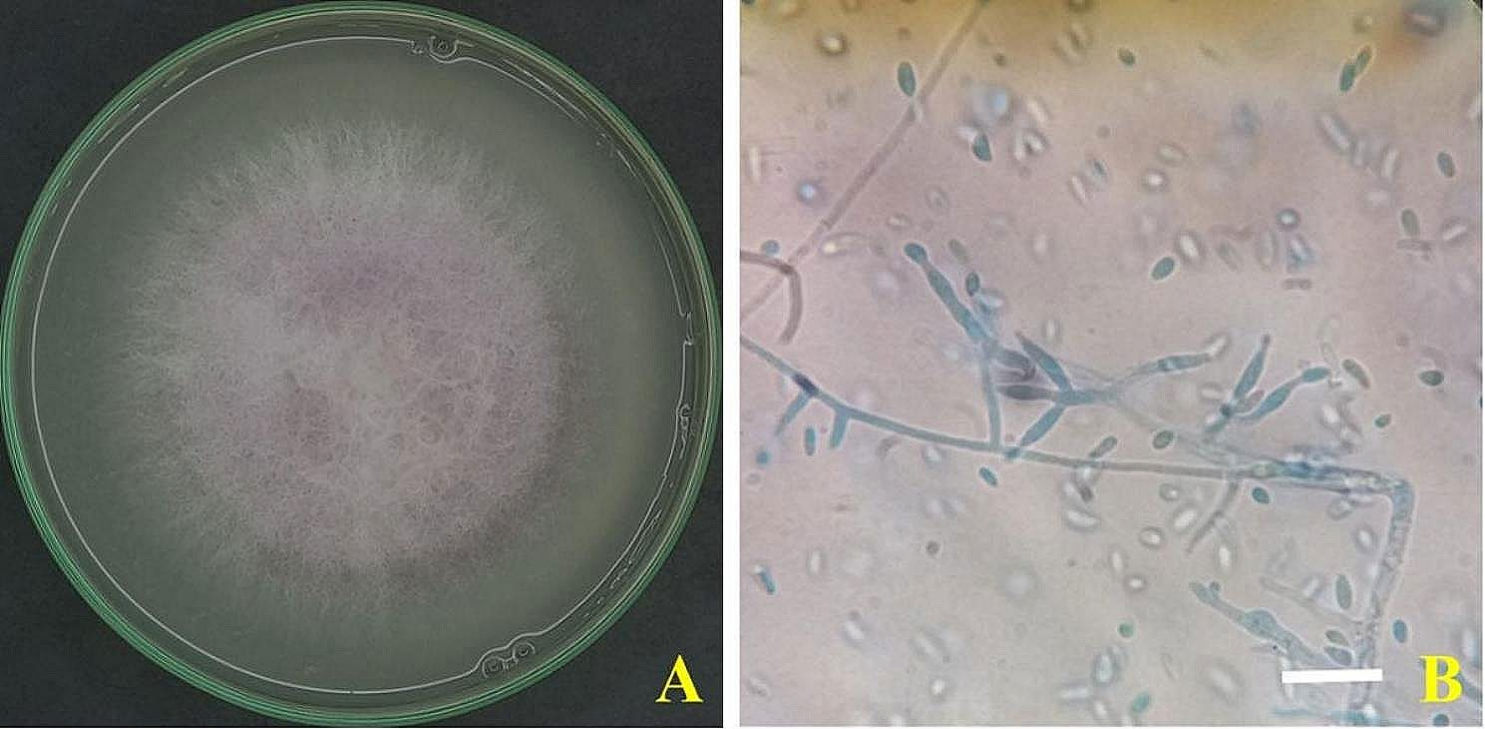
*Fusariumoxysporum* AUMC 15798. **A** 5-day-old culture on PSA at 25 °C. **B** short phialides and cylindrical conidia (Scale bar = 10 μm).

Infected onion bulbs were procured from recently harvested fields in Sohag Governorate, Egypt. The isolation of fungi from the infected onion plants yielded five *Fusarium* isolates. The five isolates had morphological characteristics of *F. oxysporum*, including fast growth rate, and violet colony color on PSA, short phialides, irregular and elliptical conidia, as well as chlamydospore production (Fig. 1).

**Fig. 2:**
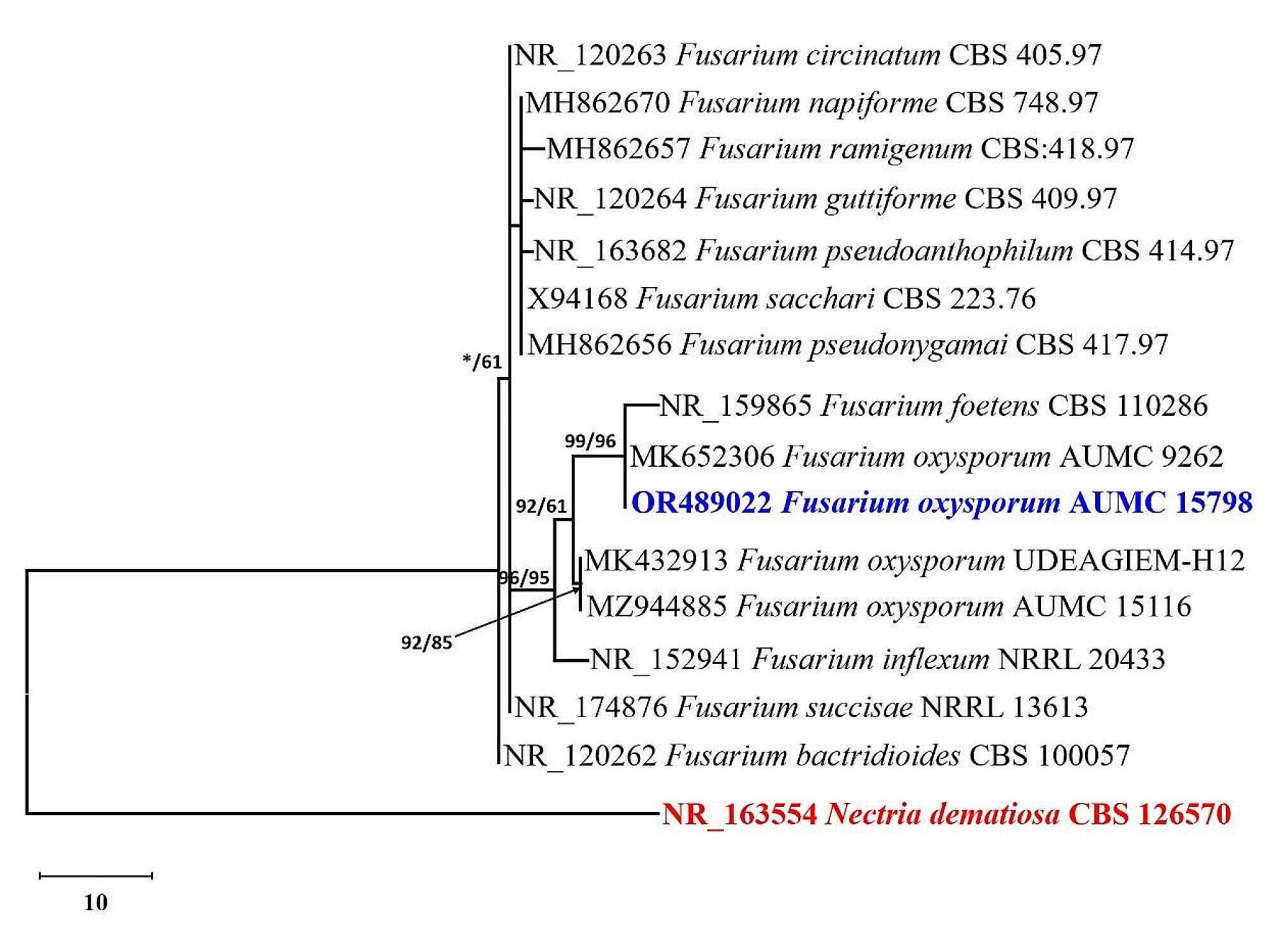
Evolutionary tree was created using the maximum parsimony analysis of the ITS sequence of *F. oxysporum* AUMC 15798 in comparison to the most related sequences of Fusarium species in GenBank (using 1000 replications of a heuristic search). Near each node, the bootstrap support values for ML/MP 50% are displayed. The Nectria dematiosa CBS 126570 outgroup is the tree’s primary node (shown in red).

The internal transcribed spacer-based sequencing (ITS) was employed to identify the most virulent isolate, which resulted in disease severity of 88.9%. The final ITS data set had 16 sequences that yielded 534 characters, of which 473 could be successfully aligned, 108 were deemed variable characters, and 9 were classified as instructive characters. With a very good support value of 99% ML/96% MP, the *Fusarium* isolate used in this investigation was assigned to the *F. oxysporum* clade and placed on the same branch as *F. oxysporum* AUMC 9262 (Fig. 2). It was determined to be *F. oxysporum*, and the ITS sequence was entered as OR489022 in GenBank.

### Pathogenicity test

In the experiment to test for pathogenicity, all isolates were used. The severity of the disease in onions caused by all five isolates ranged from 66.6 to 88.88% (Table 1).

**Table 1:** Fusarium isolates recovered from the diseased onion plants and included in the pathogenicity test

*Fusarium* isolates	Disease severity (%)
Isolate 1	88.88 ± 19.25
Isolate 2	88.88 ± 19.25
Isolate 3	73.33 ± 24.04
Isolate 4	77.77 ± 19.25
Isolate 5	66.66 ± 33.34
Control	0.00 ± 0.00
LSD (5%)	59.261

### Methanol extraction and determination of secondary metabolites

In the extraction process, it was possible to produce 32, 15, 14.95, and 45 g from *S. aromaticum*, *M. oleifera*, *A. absinthium*, and *C. procera*, respectively, from 100 g of each studied plant’s components. All of the plant sections under study had extracts that contained phenolic compounds, alkaloids, tannins, saponins, and flavonoids, with the exception of saponins, which were missing from the extract of *A. absinthium*. The current results showed that clove’s total phenolic content (240.25 mg g^−1^) is higher than what found in the other plant extracts. While the largest concentrations of alkaloids, tannins, saponins, and flavonoids were found in the seed extract of *M. oleifera* (100.5, 110.5, 180.75, and 70.25 mg g^−1^, respectively).

### Antifungal activity of the plant extracts against *F. Oxysporum* and MIC determination

The in vitro results showed that, although to varying degrees in comparison to the control, plant extracts may suppress the radial growth of the *Fusarium* pathogen. The *S. aromaticum*, *M. oleifera*, *C. procera*, and *A. absinthium*’s methanolic extract showed 63.3, 50, 46.6, and 41.1% of inhibition, respectively (Table 2).

**Table 2:** Effect of secondary metabolites of some plants on radial growth of *F. oxysporum* the causal pathogen of onion basal rot

Plant methanol extract	Colony diameter (cm)	Inhibition (%)
*A. absinthium*	5.3 ± 0.58	41.1
*C. procera*	4.8 ± 0.29	46.6
*M. oleifera*	4.5 ± 0.50	50.0
*S. aromaticum*	3.3 ± 0.58	63.3
Control	9.0 ± 0.00	0.0
LSD (5%)	1.20	

**Fig. 3:**
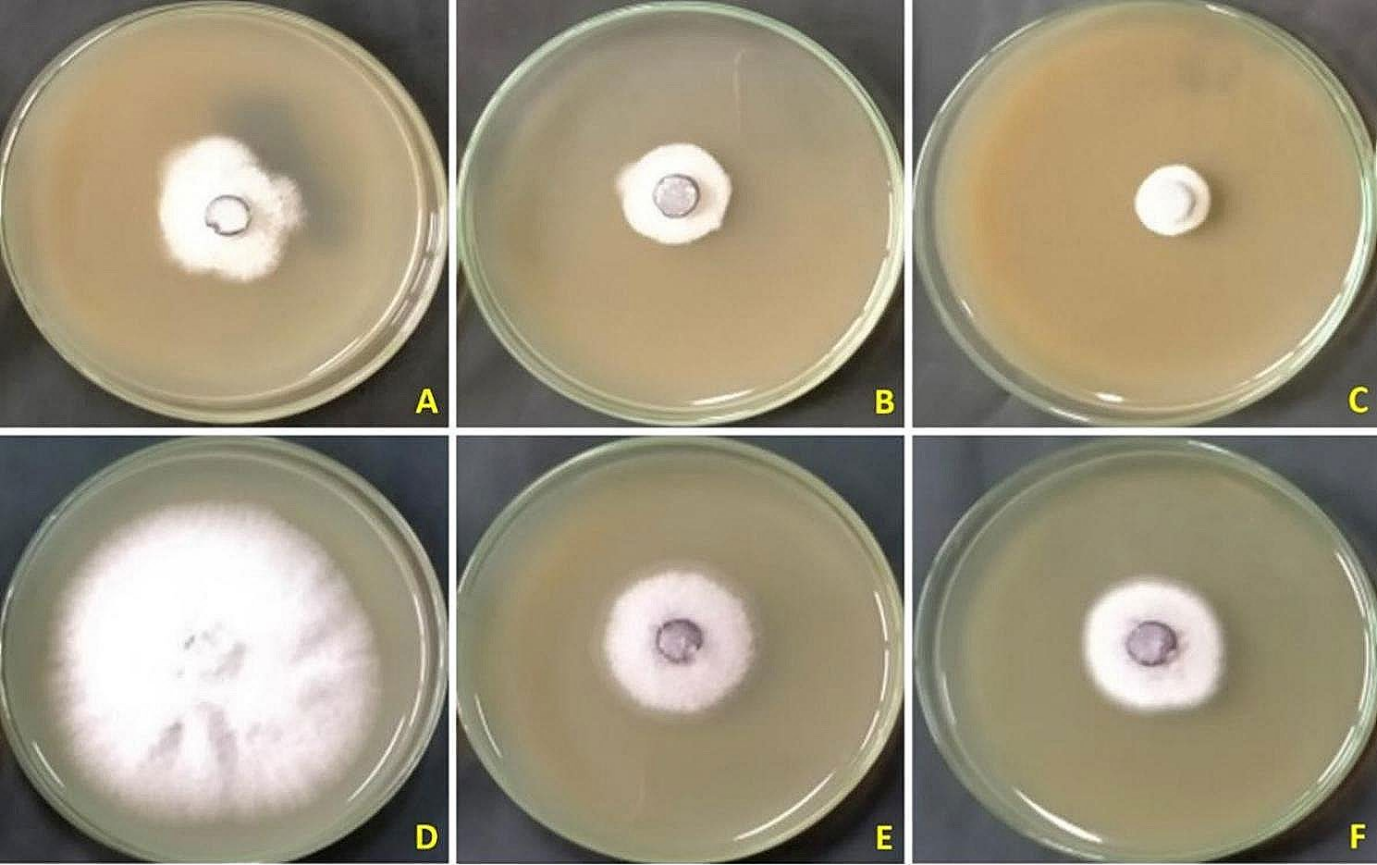
*Fusarium oxysporum* AUMC 15798’s linear growth in response to plant methanol extracts. **A–C**, *S. aromaticum* ’s methanol extract at 100, 150, and 200 ppm. **D–F**, 100, 150, and 200 ppm of *A. absinthium*  + *C. procera*  + *M. oleifera* methanol extract mixture.

Methanol extract of *S. aromaticum* with 3 concentrations, 100, 150, and 200 ppm and commixture from secondary metabolites of methanolic extracts of *M. oleifera*, *A. absinthium* and *C. procera* with three concentrations (100, 150, and 200) were assessed on radial growth of *F. oxysporum* the causal pathogen of onion basal rot. The *S. aromaticum* methanolic extract outperformed the combination of *A. absinthium*, *C. procera*, and *M. oleifera* methanolic extracts in terms of effectiveness. *F. oxysporum* was most effectively inhibited (82.2%) from growing when administered at a dose of 200 ppm. The combination of methanolic extracts from *A. absinthium*, *C. procera*, and *M. oleifera*, however, showed the lowest growth inhibition (67.7%) of *F. oxysporum* at a concentration of 200 ppm (Table 3; Fig. 3).

**Table 3:** MIC effect on *F.oxysporum* AUMC 15798’s radial growth

Treatments	Concentration (ppm)	Colony diameter (cm)	% Inhibition
*A. absinthium*	100	4.5 ± 1.73	50.0
*C. procera*	150	3.33 ± 0.58	63.0
*M. oleifera*	200	2.9 ± 0.17	67.7
*S. aromaticum*	100	2.9 ± 0.35	67.7
	150	2.16 ± 0.31	76.0
	200	1.6 ± 0.17	82.2
Control	0.00	9.0 ± 0.00	0.00
LSD (5%)		2.00	

### Biocontrol of onion basal rot disease by plant extracts in greenhouse conditions

**Fig. 4:**
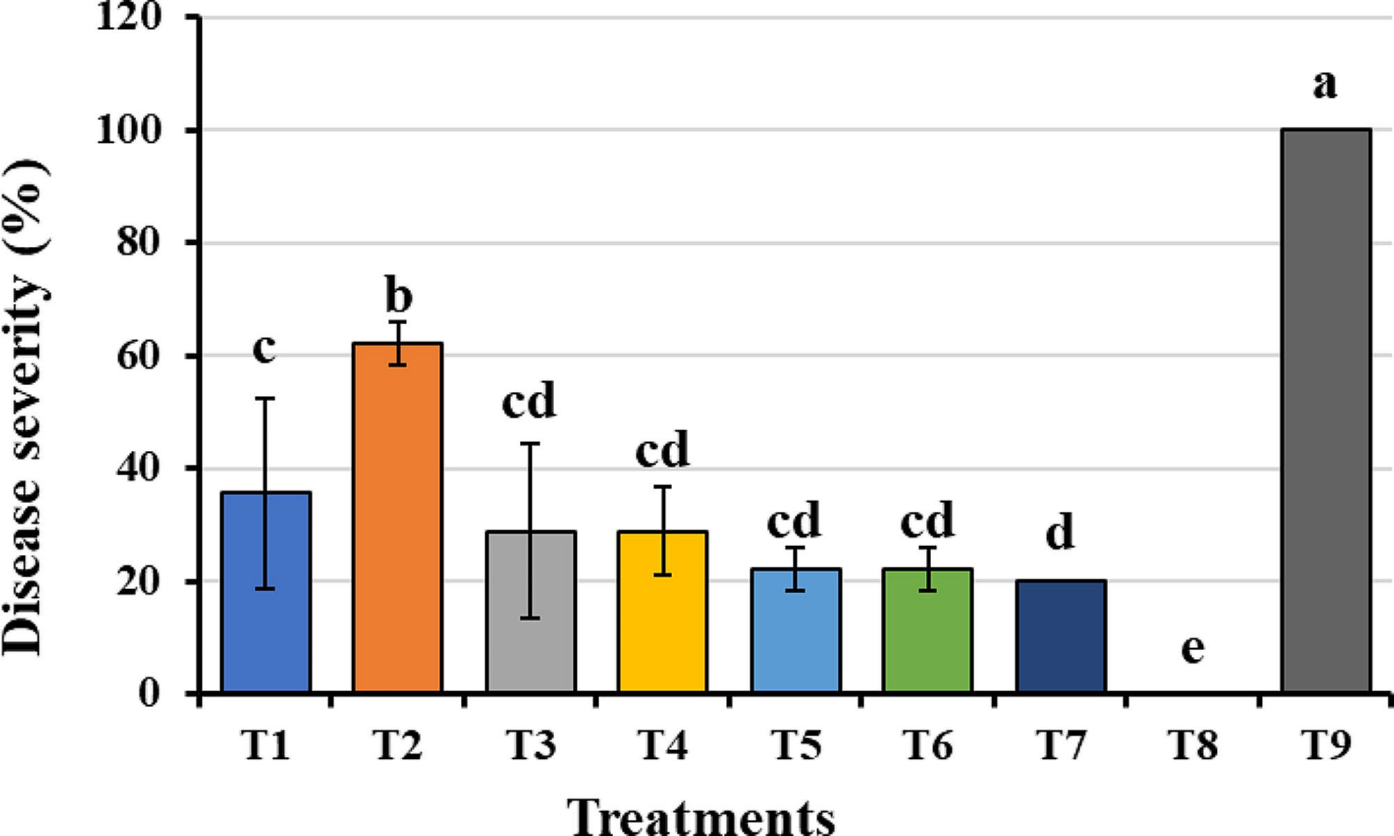
Effects of various treatments using plant methanol extracts and/or Dovex 50% control agents against *F. oxysporum* AUMC 15798-caused onion basal rot disease in greenhouse circumstances. Where: T1 = methanolic extract of *C. procera* latex; T2 = methanolic extract of *A. absinthium* leaves; T3 = methanolic extract of *M. oleifera* seeds; T4 = methanolic extract of *S. aromaticum* clove; T5 = T 1  + T 2  + T 3 + T 4; T6 = methanolic extract of Dovex 50%; T7 = methanolic extracts of Dovex 50% + T 5; T8 = negative control; T9 = positive control.

The ability of the plant extracts used in this research to prevent *F. oxysporum*, which causes onion basal rot, was tested in a greenhouse environment. Since they make the disease less severe, methanolic extracts have been demonstrated to have an effect on disease management. The disease severity was significantly reduced (20%) when using a combination of the four extracts and Dovex 50% (T7) as opposed to a combination of the four extracts (22.22 ± 3.85) or Dovex 50% alone (22.22 ± 3.85), when compared to the infected control (T9). While using the *C. procera*’s extract caused the greatest disease severity (62.22 ± 3.85) (Fig. 4).

### GC–MS analysis of ***S. aromaticum***’s methanol extract

**Fig. 5:**
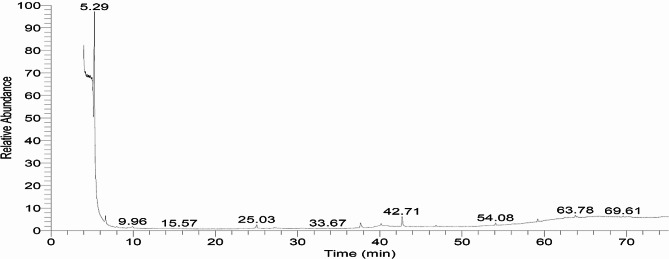
GC–MS Chromatogram of *S. aromaticum’s* methanol extract components.

The characterization of *S. aromaticum*’s methanol extract was carried out by GC–MS analysis which revealed the presence of 82 compounds which ranged from low occurrence to high abundance (Fig. 5; Table S1). Acetaldehyde, hydroxy- and 2-propanone, 1,1,3,3-tetrachloro-(42.71%), 1,2-ethanediol, and methyl alcohol (34.01%) were the main constituents detected. Also, the clove extract contained 3-allyl-6-methoxyphenyl acetate (4.1%), phenol, 2-methoxy-4-(2-propenyl)-, acetate (4.1%), and eugenol (2.25%). Some medicinally-important bioactive constituents such as propanoic acid and cyclodecasiloxane were also detected (1.0%). Some hydrocarbons were present in concentrations ranging from 0.5 to 1.5%. Peak areas for octadecane, 6-methyl undecane, tetradecane, 2,6,10-trimethyl-, dodecane, 5,8-diethyl, heptadecane, 9-hexyl-, tetradecanoic acid, 2-hydroxy-, and hexadecanoic acid, 1-(hydroxymethyl)-1,2-ethanediyl ester were all 1.41%. The presence of certain steroids (ergosta-5,22-dien-3-ol, acetate, and fatty acids (2-bromotetradecanoic acid, oleic acid) was also detected in this investigation by GC–MS (Table S1; Figure S1).

### Effect of plant extracts on the onion’s growth parameters

Onion plants’ development characteristics and morphology were estimated to be affected by the plant extracts used in this investigation. The infected control had the considerably lowest values for the parameters shoot length, root length, and bulb height, whereas the mixture of plant extracts produced the significantly highest values. The application of *S. aromaticum* methanol extract treatment produced statistically significantly longer roots (19.96 cm) than the other treatments (Table 4). The smallest root length of plants was seen under infected control. Plants treated with the mixture of plant extracts had onion neck diameters that were much larger (1.36 cm) than those of the other treatments, whereas plants in infected control had the smallest onion neck diameters (1.04 cm). The number of leaves, fresh weight, and dry weight (g.) were all statistically substantially higher in case of *S. aromaticum* methanol extract treatment than they were under the infected control (Table 4).

**Table 4:** Effect of plant extracts on the growth parameters of onion plant infected by *F. oxysporum* AUMC 15798 under greenhouse conditions

Treatments	Shoot length (cm)	Root length (cm)	Bulb height (cm)	Bulb diameter (cm)	Onion neck diameter (cm)	No. of leaves	Fresh weight (g)	Dry weight (g)
T1	52.17^ab^	14.59^bc^	3.91^a^	3.77^cd^	1.28^a^	8.67^abc^	70.00^c^	8.67^bc^
T2	46.55^ab^	9.34^d^	4.22^a^	3.21^de^	1.31^a^	9.00^ab^	68.33^c^	8.33^c^
T3	46.60^ab^	9.79^d^	3.86^a^	2.87^e^	1.23^a^	7.00^bcd^	75.00^bc^	9.67^bc^
T4	48.24^ab^	19.96^a^	3.96^a^	4.72^ab^	1.53^a^	11.00^a^	100.00^a^	14.67^a^
T5	54.36^a^	15.40^bc^	4.46^a^	4.79^a^	1.36^a^	7.33^bcd^	96.67^ab^	14.33^a^
T6	41.34^bc^	11.95^cd^	4.16^a^	3.27^de^	1.35^a^	6.33^cde^	76.67^abc^	12.67^ab^
T7	48.79^ab^	8.40^d^	4.29^a^	4.07^c^	1.36^a^	5.67^de^	73.67^bc^	12.33^abc^
T8	43.17^ab^	15.79^b^	3.67^a^	4.12^bc^	1.27^a^	5.67^de^	85.00^abc^	12.67^ab^
T9	28.9^c^	0.76^e^	2.20^a^	2.15^f^	1.04^a^	4.33^e^	25.00^d^	3.00^d^
LSD 5%	12.90	3.81	1.28	0.64	0.51	2.56	23.49	4.18

The means within each column with the same letters did not differ significantly, according to Duncan’s multiple range test at a significance level of *P =* 0.05. Where: T1 = methanolic extract of *C. procera* latex; T2 = methanolic extract of *A. absinthium* leaves; T3 = methanolic extract of *M. oleifera* seeds; T4 = methanolic extract of *S. aromaticum* clove; T5 = T_1_ + T_2_ + T_3_ + T_4_; T6 = methanolic extract of Dovex 50%; T7 = methanolic extracts of Dovex 50% + T_5_; T8 = negative control; T9 = positive control.

## Discussion

It is critically important to develop innovative, safe, and efficient strategies to control plant diseases, such as onion basal rot disease, that can be integrated into management programs for root-rot diseases. In this study, methanol extracts of *A. absinthium* leaves, *C. procera* latex, *M. oleifera* seeds, and *S. aromaticum* clove were produced, and their antifungal effects against *F. oxysporum*, the pathogen responsible for onion basal rot disease, were assessed. The methanol extract of *S. aromaticum* (clove) was identified as the powerful antifungal extract. The causative pathogen’s radial growth was inhibited to the greatest extent (63.3%) by the methanolic extract of *S. aromaticum*, followed by *M. oleifera* (50%). Numerous researchers have examined the phytoconstituents of *S. aromaticum*, and they have found that clove extracts include a wide variety of secondary metabolites. Vizhi et al. (2016) demonstrated that clove extracts in methanol and ethyl acetate contained flavonoids, phenolics, tannins, terpenoids, and steroids but did not contain alkaloids, coumarins, or quinones. Alkaloids, which are naturally occurring nitrogen-containing chemical compounds, have been found in *M. oleifera* together with flavonoids, tannins, and saponins (Adekanmi et al. 2020).

Due to its ability to yield comprehensive results and its typically sufficient identifying power compared to other techniques, GC–MS is strongly recommended for analytical determinations (Proestos and Komaitis 2013; Socrates and Mohan 2019), GC–MS analysis in the present investigation found the existence of several chemical substances which have diverse biological activities. The buildup of these substances in *S. aromaticum* is mostly connected to the metabolism of the plant. One naturally occurring, volatile organic molecule that is released from the leaves of many plant species is the highly abundant methanol (Nemecek-Marshall et al. 1995). According to research (Cossins 1964), organic acids, sugars, and amino acids like serine and methionine are the principal substrates for the incorporation of ^14^C-methanol. In several cases, the antimicrobial potentials of certain GC–MS identified substances were documented. For instance, *S. aromaticum* demonstrated observable antifungal efficacy with greater anti-candida activity than nystatin (Mansourian et al. 2014). Despite using methanol as an extraction solvent, which also contained other major chemicals, eugenol was still detected in this investigation.

In addition, 3-allyl-6-methoxyphenyl acetate was detected in the *S. aromaticum* extract, which has regarded as a potent antioxidant (Al-azem et al. 2019), and showed anti-melanogenic properties (Alam et al. 2023). Furthermore, eugenol was also detected in this analysis, a compound known for its antifungal properties (Manohar et al. 2001). According to a recent study (Mostafa et al. 2023), eugenol is identified as the primary active component in acetone and ethanol extracts of clove, demonstrating anti-candidal properties more pronounced than those of clotrimazole. The presence of phytochemicals the *S. aromaticum* clove, including alkaloids, tannins, flavonoids, anthocyanins, saponins, terpenes, steroids, and coumarins has been demonstrated (Jimoh et al. 2017). According to Cox et al. (2001), the cellular membrane appears to be the site of the primary antifungal action. The biologically-active substance cyclononasiloxane, which has hepato-protective properties (Adnan et al. 2021), was also detected from the *S. aromaticum* methanol extract.

The less abundant components in the clove’s methanol extract in this study were hydrocarbons. However, several of them have been found to have the ability to treat illnesses in both humans and animals. The investigation of Najibullah et al. (2022), which revealed the presence of hydrocarbons in related plants, lends weight to these conclusions. Additionally, the presence of a few steroids and fatty acids in this investigation was noted, with varying biological effects on lowering cholesterol and preventing heart disease. In vivo study (Venkatachalam et al. 2013), has demonstrated that propanoic acid, which detected in this study, exerted a hypoglycemic action when taken orally as it is a protein tyrosine phosphatase 1B inhibitor (PTP 1B), and maintained as anti-diabetic agent.

In this study, a combination of plant extracts and Dovex 50% decreased onion basal rot severity to 20% when compared to the infected control, while also significantly enhancing the growth and productivity of onion plants. Due to their phenolic and flavonoid components, *S. aromaticum* has been demonstrated to strongly scavenge DPPH activity in the methanol extract (Temesgen et al. 2022). Additionally, phenolic and flavonoid chemicals enhanced nutrient uptake, increased stress tolerance, and supported plant defense against pathogens and pests, among other positive effects on plant growth (Kumar et al. 2023; Pratyusha 2022).

Many plants’ vegetative growth and production have been found to be considerably enhanced by foliar application of plant extracts. In this way, using the methanol extract from *M. oleifera* improved the growth characteristics and production as well as the garlic bulb’s quality (Hegazi et al. 2016). Additionally, *Silybum marianum* plants under salt stress can grow and yield more when using Moringa leaf extract, which also boosts the production of secondary metabolites (Yap et al. 2021). Licorice root extract application greatly enhanced the physiological and biochemical characteristics, oxidative marker accumulations, antioxidant activities, nutraceutical quality, growth- and yield-related characteristics of onion cultivars (Younes et al. 2021). Garlic production and growth were stimulated by licorice root extract, and as a result the marketable bulb percentage rose while the non-marketable bulb percentage fell (Hamam et al. 2021). Wheat and barley growth has been demonstrated to be affected by *C. procera* aqueous extract in a biphasic manner, with low concentrations boosting germination and high amounts limiting growth (Radwan et al. 2019).

Amino acids, vitamins, minerals, antioxidants, enzymes, and other active components may all have a role in the positive effects of the plant extracts employed in this study. According to the study’s findings, applying a combination of plant extracts to onion plants may be a fruitful way to increase their development and output. However, additional study is required to determine the best concentration and timing for applying plant extracts as well as to explore the mechanisms by which they stimulate plant development.

In this study, it was determined if methanolic extracts of *A. absinthium* leaves, *C. procera* latex, *M. oleifera* seeds, and *S. aromaticum* clove were effective against *F. oxysporum*, the pathogen that causes onion basal rot, both in vitro and in a greenhouse setting. The extract from *S. aromaticum* exhibited the maximum inhibitory action, whereas that of *A. absinthium* achieved the lowest growth inhibition percentage. The studied methanolic extract of *S. aromaticum* contained 82 important compounds, with abundances ranging from low to high, as determined by the GC–MS analysis. When treated with a combination of plant extracts and Dovex 50%, the percentage of disease severity was significantly reduced; but, when treated with an extract of *A. absinthium*, it increased. When compared to the infected control, onion plant fresh weight and dry weight were considerably higher under the clove extract therapy. The application of the plant extracts combination was suggested as a feasible strategy for improving the growth and productivity of onion plants by the study’s findings. More research is needed to comprehend the mechanisms by which plant extracts promote plant development and to optimize the concentration and timing of administration. Therefore, we can include these plant extracts in integrated disease care to successfully avoid onion basal rot.

### Electronic supplementary material

Below is the link to the electronic supplementary material.

Supplementary Material 1.

## Data Availability

All data related to this manuscript is incorporated only in the manuscript.
