# Supplementary material for: Effectiveness of some plant extracts in biocontrol of induced onion basal rot disease in greenhouse conditions
Source: AMB Express. 2024 Jun 14;14:72. doi: 10.1186/s13568-024-01721-4 (PMC11178699; doi:10.1186/s13568-024-01721-4)
Supplement: Supplementary file 1 — Supplementary Material 1. [file 13568_2024_1721_MOESM1_ESM.pdf]

Supplementary data

Article

Effectiveness of Some Plant Methanol Extracts in Biocontrol of *Fusarium oxysporum*-Induced Onion Basal Rot Disease in Greenhouse Conditions

Mohamed G.A. Hegazy[1,*], Abdel-Raddy M. Ahmed[2], Ahmed Fathy Yousef [3], Waleed M. Ali[3], Alyaa Nasr[4], Ezzat H. Elshazly[5], Mohamed E. Shalaby[6], Islam I. Teiba[7], Osama A.M. Al-Bedak[8,*]

[1] Department of Agricultural Botany, Faculty of Agriculture, Al-Azhar University (Assiut Branch), 71524, Assiut, Egypt; mohamedhegazy.5419@azhar.edu.eg
[2] Department of Agronomy (Biochemistry), Faculty of Agriculture, Al-Azar University, (Assiut Branch), 71524, Assiut, Egypt; dr_abdelrady@yahoo.com
[3] Department of Horticulture, Faculty of Agriculture, University of Al-Azhar (Assiut Branch), 71524, Assiut, Egypt; ahmed.yousuf@azhar.edu.eg; waleed.mohmmed@azhar.edu.eg
[4] Botany & Microbiology Department, Faculty of Science, Menoufia University, Shebin Elkom, 32511 Egypt; alyaa.abd@science.menofia.edu.eg
[5] Botany & Microbiology Department Faculty of Science, Al-Azhar University (Assiut Branch), 71524, Assiut, Egypt; ezzathamdy83@azhar.edu.eg
[6] Department of Plant Production, Faculty of Agriculture (Saba Basha), Alexandria University, Alexandria 21531, Egypt; m-shalaby@alexu.edu.eg
[7] Microbiology, Botany Department, Faculty of Agriculture, Tanta University, 31527, Tanta, Egypt; islam.tayba@agr.tanta.edu.eg
[8] Assiut University Mycological Centre, Assiut, 71511, Egypt; osamaalbedak@science.au.edu.eg
*Correspondences: osamaalbedak@science.au.edu.eg - mohamedhegazy.5419@azhar.edu.eg

Table S1: GC-MS identified bioactive compounds in *S. aromaticum*

No.	RT	Compound Name	Molecular Formula	Molecular Weight	Area (%)
1	4.27	Hydroxylamine	H_3NO	33	0.10
2	4.66	Silane	H_4Si	32	0.17
3	5.06	1,2-Ethanediol (Ethylene Glycol)	$C_2H_6O_2$	62	34.01
4	5.06	Methyl Alcohol	CH_4O	32	34.01
5	5.29	Acetaldehyde, hydroxy-	$C_2H_4O_2$	60	42.71
6	5.29	2-Propanone, 1,1,3,3-tetrachloro-	$C_3H_2C_{14}O$	194	42.71
7	6.03	Hydroxyacetic acid, hydrazide	$C_2H_6N_2O_2$	90	0.13
8	6.67	Acetic acid	$C_2H_4O_2$	60	2.37
9	6.67	Hydrazine, ethyl-	$C_2H_8N_2$	60	2.37
10	6.67	Tetraacetyl-d-xylonic nitrile	$C_{14}H_{17}NO_9$	343	2.37

12	7.49	14-Formol-9-hydroxyarteether	$C_{17}H_{26}O_7$	342	0.18
13	7.49	Gibb-3-ene-1,10-dicarboxylic acid, 2,4a-dihydroxy-1-methyl-8-methylene-, 1,4a-lactone, 10-methyl ester, (1à,2á,4aà,4bá,10á)-(Gibberillic acid)	$C_{20}H_{24}O_5$	344	0.18
14	8.00	Pregna-5,8-diene-3á,11à-diol-20-one	$C_{25}H_{34}O_5$	414	0.6
15	8.00	2,6-dibromo-4-(4-morpholinylthiocarbonyl)phenyl ester	$C_{13}H_{13}Br_2NO_3S$	421	0.60
16	8.00	Pregna-6,16-diene-11,20-diol, 3,9-epoxy-18-[N-methyl-N-[14-(2'-epoxyethyl)]amino]-	$C_{25}H3_7NO_5$	431	0.60
17	8.00	1-Nitro-á-d-arabinofuranose, tetraacetate	$C_{13}H_{17}NO_{11}$	363	0.60
18	8.00	1-Nitro-2-acetamido-1,2-dideoxy-d-glucitol	$C_8H_{16}N_2O_7$	252	0.60
19	9.96	Cyclopropanebutanoic acid, 2-[[2-[[2-[(2-pentylcyclopropyl)methyl] cyclopropyl]methyl]cyclopropyl]methyl]-, methyl ester	$C_{25}H_{42}O_2$	374	0.96
20	9.96	3-(2,5,8,11,14-Pentaoxacyclohexadecyl)-1,5,8,11,14,17-hexooxacyclononadecane	$C_{24}H_{46}O_{11}$	510	0.96
21	9.96	Tetradecanoic acid, 2-hydroxy-	$C_{14}H_{28}O_3$	244	0.96
22	9.96	Salmeterol	$C_{25}H_{37}NO_4$	415	0.96
23	9.96	Cholesta-8,24-dien-3-ol, 4-methyl-, (3á,4à)-	$C_{28}H_{46}O$	398	0.96
24	12.32	Glycine, N-[(3à,5á,7à,12à)-24-oxo-3,7,12-tris[(trimethylsilyl)oxy]cholan-24-yl]-, methyl ester	$C_{36}H_{69}NO_6Si_3$	695	0.20
25	12.32	4,25-Secoobscurinervan-4-one, O-acetyl-22-ethyl-15,16-dimethoxy-, (22à)-	$C_{27}H_{36}N_2O_6$	484	0.20
26	12.32	Milbemycin B, 6,28-anhydro-15-chloro-25-isopropyl-13-dehydro-5-O-demethyl-4-methyl-	$C_{33}H_{47}ClO_7$	590	0.20
27	12.32	2-Formamido-2',4'-dinitro-4-[trifluoromethyl]-diphenylsulfide	$C_{14}H_8F_3N_3O_5S$	387	0.20
28	12.32	9-Octadecenoic acid (Z)-, 2-butoxyethyl ester	$C_{24}H_{46}O_3$	382	0.20
29	25.03	Octadecane, 6-methyl-	$C_{19}H_{40}$	268	1.41
30	25.03	Undecane	$C_{11}H_{24}$	156	1.41
31	25.03	Tetradecane, 2,6,10-trimethyl-	$C_{17}H_{36}$	240	1.41
32	25.03	Dodecane, 5,8-diethyl-	$C_{16}H_{34}$	226	1.41
33	25.03	Heptadecane, 9-hexyl-	$C_{23}H_{48}$	324	1.41
34	27.15	Calconcarboxylic acid	$C_{21}H_{14}N_2O_{7S}$	438	0.37
35	27.15	Dihydroartemisinin, 9-deoxy-9-[2-(isopropylaminocarbonyl)ethyl]-	$C_{21}H_{35}NO_5$	381	0.37
36	27.15	8H-Azecino[5,4-b]indol-8-one, 5-ethylidene-1,2,3,4,5,6,7,9-octahydro-6-(2-hydroxyethyl)-3-methyl-, [S-(E)]-	$C_{20}H_{26}N_2O_2$	326	0.37
37	27.15	Pregan-20-one, 2-hydroxy-5,6-epoxy-15-methyl-	$C_{22}H_{34}O_3$	346	0.37
38	27.15	Pregnane-3,11,20,21-tetrol, cyclic20,21-(butyl boronate), (3à,5á,11á,20R)-	$C_{25}H_{43}BO_4$	418	0.37
39	27.15	Cyclopentanone, 2-(2-octenyl)-	$C_{13}H_{22}O$	194	0.14
40	37.36	Phenol, 2-methoxy-3-(2-propenyl)-	$C_{10}H_{12}O_2$	164	2.25

41	37.66	3-Allyl-6-methoxyphenol	$C_{10}H_{12}O_2$	164	2.25
42	37.66	Eugenol	$C_{10}H_{12}O_2$	164	2.25
43	37.66	Phenol, 2-methoxy-4-(1-propenyl)-	$C_{10}H_{12}O_2$	164	2.25
44	39.39	6,7-Epoxypregn-4-ene-9,11,18-triol-3,20-dione, 11,18-diacetate	$C_{25}H_{32}O_8$	460	0.39
45	39.39	6-Methyl-11-propenyl-5-(toluene-4-sulfonyloxy)-12,13-dioxatricyclo[7.3.1.0(1,6)]tridecane-8-carboxylic acid, methylester	$C_{24}H_{32}O_7S$	464	0.39
46	39.39	Vanillin lactoside	$C_{20}H_{28}O_{13}$	476	0.39
47	39.39	10,13-Dioxatricyclo[7.3.1.0(4,9)]tridecan-5-ol-2-carboxylic acid, 4-methyl-11-(1-propenyl)-, methyl ester	$C_{17}H_{26}O_5$	310	0.39
48	39.39	4-methyl-11-(1-propenyl)-, methyl ester Androstan-3-one, cyclic 1,2-ethanediyl mercaptole, (5à)-	$C_{21}H_{34}S_2$	350	0.39
49	40.17	2-[4-methyl-6-(2,6,6-trimethylcyclohex-1-enyl)hexa-1,3,5-trienyl]cyclohex-1-en-1-carboxaldehyde	$C_{23}H_{32}O$	324	0.83
50	40.17	Butyl 6,9,12,15-octadecatetraenoate	$C_{22}H_{36}O_2$	332	0.83
51	40.17	Ethyl 6,9,12,15-octadecatetraenoate	$C_{20}H_{32}O_2$	304	0.83
52	40.75	2-Methyl-E,E-3,13-octadecadien-1-ol	$C_{19}H_{36}O$	280	0.23
53	40.57	Gibberellic acid	$C_{19}H_{22}O_6$	346	0.23
54	40.57	6-Aminohexanamide, N-methyl-N-[4-(1-pyrrolidinyl)-2-butynyl]-N'-[2-aminobutanoyl]-	$C_{18}H_{32}N_4O_2$	336	0.23
55	40.85	Ergosta-5,22-dien-3-ol, acetate,	$C_{30}H_{48}O_2$	440	0.20
56	41.49	Ethyl iso-allocholate	$C_{26}H_{44}O_5$	436	0.10
57	41.49	n-Butyl ricinoleate	$C_{22}H_{42}O_3$	354	0.10
58	42.71	3-Allyl-6-methoxyphenyl acetate	$C_{12}H_{14}O_3$	206	4.10
59	42.71	Phenol, 2-methoxy-4-(2-propenyl)-, acetate	$C_{12}H_{14}O_3$	206	4.10
60	46.83	4-Piperidineacetic acid, 1-acetyl-5-ethyl-2-[3-(2-hydroxyethyl)- 1H-indol-2-yl]-à-methyl-, methyl ester	$C_{23}H_{32}N_2O_4$	400	0.47
61	46.83	Hexadecanoic acid, 1-(hydroxymethyl)-1,2-ethanediyl ester	$C_{35}H_{68}O_5$	568	0.47
62	46.83	3-Pyridinecarboxylic acid, 2,7,10-tris(acetyloxy)-1,1a,2,3,4,6,7,10, 11,11a-decahydro-1,1,3,6,9-pentamethyl-4-oxo-4a,7a-epoxy-5H-cyclopenta[a]cyclopropa[f]cycloundecen-11-ylester, (pyridine alkaloid)	$C_{32}H_{39}NO_{10}$	597	0.47
63	54.08	Cyclononasiloxane, octadecamethyl-	$C_{18}H_{54}O_9Si_9$	666	0.98
64	58.32	Oleic Acid	$C_{18}H_{34}O_2$	282	0.20
65	58.32	Oleic acid, 3-(octadecyloxy)propylester	$C_{39}H_{76}O_3$	592	0.20
66	59.20	Cyclodecasiloxane, eicosamethyl-	$C_{20}H_{60}O_{10}Si_{10}$	740	1.00
67	59.20	1-Monolinoleoylglycerol trimethylsilylether	$C_{27}H_{54}O_4Si_2$	498	1.00
68	59.20	Propanoic acid, 2-(3-acetoxy-4,4,14-trimethylandrost-8-en-17-yl)-	$C_{27}H_{42}O_4$	430	1.00
69	59.20	(5á)Pregnane-3,20á-diol, 14à,18à-[4-methyl-3-oxo-(1-oxa-4-aza	$C_{28}H_{43}NO_6$	489	1.00

		butane-1,4-diyl)]-, diacetate			
70	59.20	9,12,15-Octadecatrienoic acid, 2,3-bis[(trimethylsilyl)oxy]propylester, (Z,Z,Z)-	$C_{27}H_{52}O_4Si_2$	496	1.00
71	60.08	9-Octadecenoic acid (Z)-, tetradecylester	$C_{32}H_{62}O_2$	478	0.14
72	60.08	9-Octadecenoic acid, 1,2,3-propanetriylester, (E,E,E)-	$C_{57}H_{104}O_6$	884	0.14
73	62.46	5H-Cyclopropa[3,4]benz[1,2-e]azulen-5-one, 3,9,9a-tris(acetyloxy)-3-[(acetyloxy)methyl]-2-chloro-1,1a,1b,2,3,4,4a,7a,7b,8, 9,9a-dodecahydro-4a,7b-dihydroxy-1,1 ,6,8-tetramethyl-, [1aR-(1aà,1bá,2à,3á,4aá,7aà,7bà,8à,9á, 9aà)]-	$C_{28}H_{37}ClO_{11}$	584	0.23
74	62.46	8,14-Seco-3,19-epoxyandrostane-8,14-dione, 17-acetoxy-3á-methoxy-4,4-dimethyl-	$C_{24}H_{36}O_6$	420	0.23
75	62.92	Oleic acid, eicosyl ester	$C_{38}H_{74}O_2$	562	0.28
76	63.79	7,8-Epoxylanostan-11-ol, 3-acetoxy-	$C_{32}H_{54}O_4$	502	0.88
77	63.79	3,5,9-Trioxa-5-phosphaheptacos-18-en-1-aminium, 4-hydroxy-N,N,N-trimethyl-10-oxo-7-[(1-oxo-9-octadecenyl)oxy]-, hydroxide, inner salt, 4-oxide, (R)-	$C_{44}H_{84}NO_8P$	785	0.88
78	63.79	5H-Cyclopropa[3,4]benz[1,2-e]azulen-5-one, 3,9,9a-tris(acetyloxy)-3-[(acetyloxy)methyl]-2-chloro-1,1a,1b,2,3,4,4a,7a,7b,8, 9,9a-dodecahydro-4a,7b-dihydroxy-1,1,6,8-tetramethyl-, [1aR-(1aà,1bá,2à,3á,4aá,7aà,7bà,8à,9á, 9aà)]-	$C_{28}H_{37}ClO_{11}$	584	0.88
79	63.79	Docosanoic acid, 1,2,3-propanetriylester	$C_{69}H_{134}O_6$	1058	0.88
80	69.61	.psi.,.psi.-Carotene, 1,1',2,2'-tetrahydro-1,1'-dimethoxy-	$C_{42}H_{64}O_2$	600	0.53
81	70.02	9-Hexadecenoic acid, 9-octadecenylester, (Z,Z)-	$C_{34}H_{64}O_2$	504	0.14
82	70.08	Dasycarpidan-1-methanol, acetate (ester)	$C_{20}H_{26}N_2O_2$	326	0.12

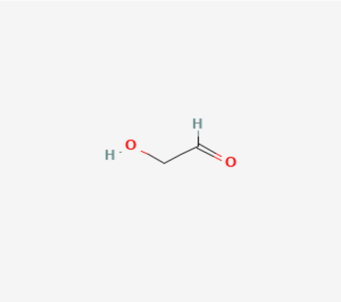

Acetaldehyde, hydroxy-

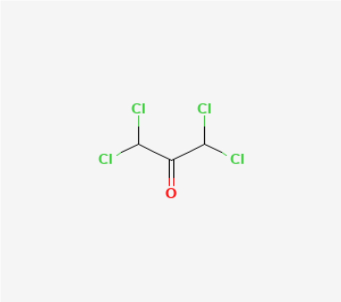

2-propanone, 1,1,3,3-tetrachloro-

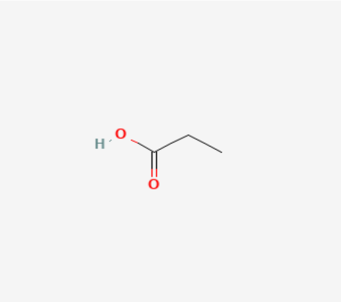

Propanoic acid

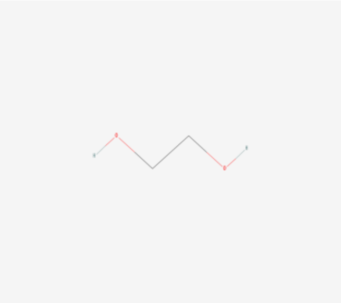

1,2-Ethanediol

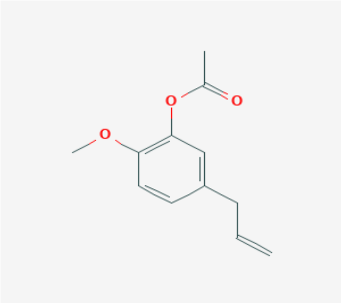

3-Allyl-6-methoxyphenyl acetate

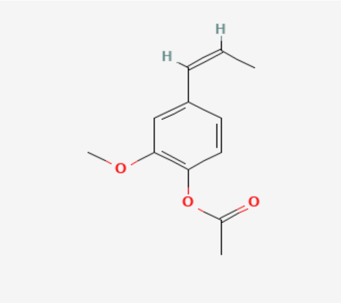

Phenol, 2-methoxy-4-(2-propenyl)-, acetate

Figure S1: The depiction of some major compounds in the methanol extract of *S. aromaticum* as detected by GC-MS analysis *(Information, 2023)*

Information, N. C. f. B. (2023). PubChem Compound Summary for CID 1715136, Phenol, 2-methoxy-4-(1-propenyl)-, acetate, (Z)-. . Retrieved from https://pubchem.ncbi.nlm.nih.gov/compound/1715136.
